# CD44 mediates the internalization of foot-and-mouth disease virus through macropinocytosis

**DOI:** 10.1186/s13567-025-01555-3

**Published:** 2025-06-21

**Authors:** Xuefei Wang, Hu Dong, Jin’en Wu, Yun Zhang, Shiqi Sun, Huichen Guo

**Affiliations:** 1 State Key Laboratory for Animal Disease Control and Prevention, College of Veterinary Medicine, Lanzhou University, Lanzhou Veterinary Research Institute, Chinese Academy of Agricultural Sciences, Lanzhou, 730000 China; 2 State Key Laboratory for Animal Disease Control and Prevention, Harbin Veterinary Research Institute, Chinese Academy of Agricultural Sciences, Harbin, China

**Keywords:** FMDV, CD44, macropinocytosis, internalization

## Abstract

Foot-and-mouth disease virus (FMDV), a member of the Picornavirus family, poses a significant threat to global animal husbandry. While it is known that macropinocytosis plays a crucial role in the entry of FMDV into cells, the specific cellular proteins that regulate this process and the downstream signalling pathways associated with these proteins are not yet fully understood. In this study, we demonstrated that the membrane protein cluster of differentiation 44 (CD44) is essential for the internalization of FMDV in BHK-21 cells, while it is not involved in the attachment of the virus to the cell. In addition, we found that CD44 is internalized in response to FMDV entry. Interestingly, the use of a macropinocytosis inhibitor impaired both CD44 internalization and FMDV entry and infection, indicating a strong connection between these two processes. Further experiments revealed that CD44 facilitates FMDV internalization through macropinocytosis. Importantly, our study shows that CD44 interacts with FMDV capsid proteins VP2 and VP3 and becomes phosphorylated during the entry of the virus. The phosphorylation of CD44 subsequently promotes the phosphorylation of p21-activated kinase 1 (PAK1), which is a critical component in the macropinocytotic entry of FMDV. Overall, this study provides new insights into the role of CD44 in the invasion of pathogens into host cells and highlights potential strategies for improving FMDV vaccines.

**Supplementary Information:**

The online version contains supplementary material available at 10.1186/s13567-025-01555-3.

## Introduction

Macropinocytosis differs from other pinocytic pathways, such as clathrin-mediated endocytosis (CME), caveolin-mediated endocytic pathway, and lipid raft-mediated endocytosis. It typically begins with the phosphorylation of specific membrane proteins, which activates intracellular signalling cascades. This activation leads to the rearrangement of actin and the formation of irregular ruffles and blebs. These irregular ruffles then collapse toward the cell membrane, enveloping the virus-receptor complex along with the dissociated virus and other fluid-phase macromolecules [1–4].

Eventually, these structures pinch off from the cell membrane and enter the cytoplasm as large (0.5–10 μm) and irregularly shaped endocytic vesicles known as macropinosomes [2–4]. The macropinocytotic entry of FMDV requires the involvement of various proteins, including Rho GTPases (Rac1), Na^+^/H^+^ exchangers, kinases (PAK1 and protein kinase C), myosin II, dynamin-2, and receptor tyrosine kinases [5]. However, the remaining signalling molecules that regulate viral macropinocytotic entry have yet to be fully identified.

CD44 is a type I cell surface glycoprotein transmembrane receptor that plays a vital role in interactions between cells and the cell–matrix. It primarily interacts with several molecules within the extracellular matrix, most notably hyaluronic acid [6, 7]. CD44 is widely expressed in various organs, including the thymus, lymphoid organs, heart, liver, spleen, lung, and kidney [8]. It is involved in the regulation of numerous critical signalling pathways, thus contributing to several physiological and pathological processes such as cell migration [9, 10], adhesion [11–13], and proliferation [14].

Moreover, CD44 has been identified as a marker for cancer-initiating cells, also known as cancer stem cells, in various malignancies of haematopoietic and epithelial origins [15]. Additionally, CD44 plays essential roles in inflammatory and immune responses, aiding in the ingestion and clearance of particles and apoptotic cells [16–18]. It also negatively regulates cell autophagy by decreasing levels of PIK3R4 and PIK3C3 and disrupting STAT3-dependent PtdIns3K complexes in vascular endothelial cells [19]. Furthermore, CD44 is involved in the regulation of glucose and lipid homeostasis [20].

One of CD44’s critical functions is its participation in the infection and propagation of pathogens. For instance, the parasite *Plasmodium falciparum* utilises CD44 as a co-receptor for invading erythrocytes [21], while Group A *Streptococcus* utilises CD44-mediated cell signalling to facilitate tissue invasion [22]. In recent decades, studies have shown that CD44 is associated with FMDV infection. For example, a quantitative proteomic analysis indicated that the CD44 protein level is significantly upregulated in response to FMDV infection [23]. Additionally, CD44 is linked to T-cell responses following FMD vaccine inoculation in Chinese Holstein cows [24].

Interestingly, CD44 participates in processes associated with macropinocytosis in some pathogen-infected cells, such as cytoskeletal rearrangement and membrane ruffling. Specifically, CD44-dependent binding of Group A *Streptococcus* to polarised monolayers of human keratinocytes has been shown to induce significant cytoskeletal rearrangements, resulting in membrane ruffling and disruption of intercellular junctions [22]. However, as a membrane signalling molecule, the extent to which CD44 influences FMDV macropinocytotic entry and the specifics of CD44-mediated downstream signalling remain to be fully understood.

We demonstrated that CD44 interacts with FMDV VP2 and VP3 and undergoes phosphorylation during FMDV entry. Activated CD44 subsequently initiated the activation of PAK1, a crucial component in FMDV macropinocytotic entry, promoting macropinocytosis.

## Materials and methods

### Cells, viruses, and infection

BHK-21 (baby hamster kidney; American Type Culture Collection [ATCC] CCL-10) and PK-15 cells (porcine kidney; ATCC CCL-33) were cultured in Dulbecco’s Modified Eagle’s Medium (DMEM) (Gibco, California, USA), which was supplemented with 10% fetal bovine serum (FBS) (Gibco), penicillin (100 U/mL), and streptomycin (100 mg/mL) (Gibco). The cells were incubated at 37 °C in a 5% CO_2_ atmosphere.

The FMDV serotype O strain O/China/99 (GenBank accession no. AF506822.2) was maintained by the OIE/National Foot-and-Mouth Disease Reference Laboratory (Lanzhou, China). FMDV was propagated in BHK-21 cells, and the viral titres were measured using a 50% tissue culture infective dose (TCID_50_) assay in BHK-21 cells.

### Antibodies and reagents

Rabbit anti-CD44 and rabbit anti-PAK1 monoclonal antibodies were obtained from Abcam (Cambridge, MA, USA). Mouse anti-Flag monoclonal antibody and rabbit anti-HA polyclonal antibody were sourced from Proteintech (Wuhan, China). Rabbit anti-pPAK1 (Thr423) monoclonal antibody was purchased from Thermo Fisher Scientific (Waltham, MA, USA). Mouse anti-actin monoclonal antibody was purchased from CWBIO (Beijing, China).

The anti-FMDV structural protein polyclonal pig antiserum was prepared in our laboratory by immunising pigs with FMDV (O/BY/CHA/2010; GenBank accession no. JN998085.1) VLP. Secondary antibodies conjugated with horseradish peroxidase (HRP), fluorescein isothiocyanate (FITC), tetramethyl rhodamine isocyanate (TRITC) and Alexa Fluor 647 were purchased from Sigma-Aldrich (St. Louis, MO, USA). Lipofectamine RNAiMAX and Lipofectamine 2000 were obatined from Invitrogen (California, USA).

CPZ, MβCD, and EIPA were purchased from MedChemEx Press (Monmouth Junction, New Jersey, USA). Additionally, FITC-Dextran was acquired from Sigma-Aldrich (St. Louis, MO, USA) and Alexa Fluor 488-phalloidin was sourced from Thermo Fisher Scientific (Waltham, MA, USA).

### Plasmid constructs

The cDNAs were amplified from BHK-21 cells or PK-15 cells using RT-PCR and subsequently subcloned into the pCMV-C-HA vector (Beyotime Biotechnology, Shanghai, China) to create HA-tagged CD44 plasmids. structural proteins of FMDV, namely VP0, VP1, and VP3, were previously constructed in our laboratory [25, 26]. Additionally, Tsingke (Beijing, China) synthesised the EGFP-tagged Rabankyrin-5. All DNA constructs were verified through sequencing.

### RNA interference

For RNA interference (RNAi), siRNAs that target candidate genes and a negative-control (NC) siRNA were synthesised by GenePharma (Shanghai, China). The three sequences of the siRNAs for hamster CD44 are as follows: 5′-GGACCAGUUACCAUAACUATT-3′, 5′-GACUGAUGGAUCCAAAUUATT-3′, and 5′-CACCUACCUUCCCACUAUATT-3′. The sequence of the NC siRNA is 5′-UUCUCCGAACGUGUCACGU-3′.

### Transfection of siRNA and plasmids

Each siRNA was transfected into cells at a final concentration of 10 nM using the Lipofectamine RNAiMAX reagent (Invitrogen). After 36 h post-transfection (hpt), the cells were infected with FMDV and analysed using RT-qPCR, western blot, or indirect immunofluorescence analysis. For plasmid transfection, the cells were transfected with the corresponding plasmids using Lipofectamine 2000. After 24 h post-transfection, the cells were also infected with FMDV and subjected to RT-qPCR, western blot or indirect immunofluorescence analysis.

### Viral adsorption assay

The adsorption assay was performed using a modified version of a previously established method [27]. In summary, cells were transfected with plasmids encoding HA-EV and HA-CD44, or with NC siRNA and siRNA targetting CD44. 24 or 36 h post-transfection, the cells were infected with FMDV (10 MOI) at 4 ℃ for 1 h. After infection, the cells were washed three times with cold PBS and subjected to RT-qPCR analysis.

### Viral internalization assay

The internalization assay was performed following a previously established method with some modifications [27]. In brief, cells were transfected with plasmids encoding HA-EV and HA-CD44, or with NC siRNA and siRNA targetting CD44. 24- or 36-h post-transfection, the cells were infected with FMDV (10 MOI) at 4 ℃ for 1 h. After infection, the cells were washed three times with cold PBS to remove any unbound particles and then incubated at 37 ℃ for either 30 min or 1 h. Following this incubation, the cells were treated with either trypsin or proteinase K (1 mg/mL) on ice to eliminate non-internalized particles before undergoing RT-qPCR.

### Quantitative real-time PCR

RNAiso Plus (TaKaRa) was used to extract RNA from PK-15 cells and BHK-21 cells. A total of 1000 ng of RNA was then converted into cDNA using the 5 × RT Master Mix (TaKaRa) following the manufacturer’s instructions for reverse transcription. The resulting cDNAs were subjected to qPCR using an Applied Biosystems 7500 real-time PCR system. The specific primers for each gene were as follows: FMDV 3D, 5′-CAAACCTGTGATGGCTTCGA-3′ and 5′-CCGGTACTCGTCAGGTCCA-3′; porcine GAPDH, 5′-ACATGGCCTCCAAGGAGTAAGA-3′ and 5′-GATCGAGTTGGGGCTGTGACT-3′; mouse GAPDH, 5′-AAGAAGGTGGTGAAGCAGGCATC-3′ and 5′-CGGCATCGAAGGTGGAAGAGTG-3′.

### Western blot analysis

Cells were lysed to extract the total protein fraction. The proteins were denatured using 1 × SDS loading buffer, separated by SDS-PAGE, and transferred to NC membranes. The membranes were blocked for 1 h with 5% skim milk and then incubated overnight with primary antibodies. Afterwards, the membranes were washed five times with Tris-buffered saline-Tween (TBST) and incubated for 1 h with HRP-conjugated secondary antibodies, followed by another five washes with TBST. Finally, the membranes were incubated with an enhanced chemiluminescence detection reagent (Thermo Fisher Scientific, Inc., Rockford, IL, USA) to visualise the protein bands.

### Virus titration

Virus infectivity was titrated by endpoint dilution. Serially diluted samples were utilised to infect the specified cells in 96-well plates, and the TCID_50_ was determined using the Reed-Muench method.

### FITC-dextran uptake assays

BHK-21 cells were pre-chilled at 4 ℃ for 1 h and then either mock-infected or infected with FMDV (10 MOI) at 4 ℃ for 1 h. Following the infection, the cells were washed three times with cold PBS to remove any unbound particles. The inoculum was then replaced with medium containing FITC-dextran 10 K (2 mg/mL) and incubated at 37 °C for a specified period. After incubation, the cells were washed three times with cold PBS and then twice with a low pH buffer (0.1 M sodium acetate, 0.05 M NaCl, pH 5.5). Finally, the cells were fixed in 4% paraformaldehyde and prepared for indirect immunofluorescence analysis.

### Indirect immunofluorescence

BHK-21 cells that were transfected and/or infected were fixed using 4% paraformaldehyde for 15 min at 37 °C. After fixing, the cells were washed three times with cold PBS. To permeabilise the fixed cells, they were treated with 0.1% Triton X-100 in PBS for 10 min. The cells were then blocked with 5% bovine serum albumin in PBS for 1 h.

Next, the cells were incubated overnight at 4 °C with specific primary antibodies. After this incubation, they were washed three times with PBS and then incubated for 1 h at 37 °C with the corresponding secondary antibody. F-actin was stained using Alexa Fluor 488 phalloidin, and the nuclei were stained with DAPI (Beyotime Biotechnology, Shanghai, China) for 10 min. Images were captured using a laser scanning confocal microscope (Leica SP8; Leica, Solms, Germany).

### Immunoprecipitation assay

BHK-21 cells that were either transfected with specific plasmids or infected with FMDV were lysed on ice for 1 h. The lysates were then centrifuged at 15 000 × *g* for 20 min at 4 °C. Cell debris was discarded, and the supernatants were immunoprecipitated with specific antibodies overnight at 4 °C. The immune complexes were subsequently incubated with protein G-agarose beads (GE Healthcare, Chicago, IL, USA) for 2 h. After incubation, the beads were washed five times with lysis buffer and then eluted in SDS-PAGE buffer. Finally, the samples were subjected to western blot analysis.

### Molecular docking

The crystal structure of FMDV was obtained from PDB (ID: 7ENO), and the secondary structure of CD44 was sourced from PDB (ID: A0A1U7Q2F4). Their interactions were simulated using the server’s default parameters.

### Statistical analysis

Data were collected from at a minimum of three independent experiments for quantitative analysis, and results are presented as means ± SD. All statistical analyses were conducted using either a *t*-test or one-way analysis of variance. A *P*-value of < 0.05 was considered to indicate a significant difference.

## Results

### CD44 is required for FMDV internalization in BHK-21 cells

**Figure 1:**
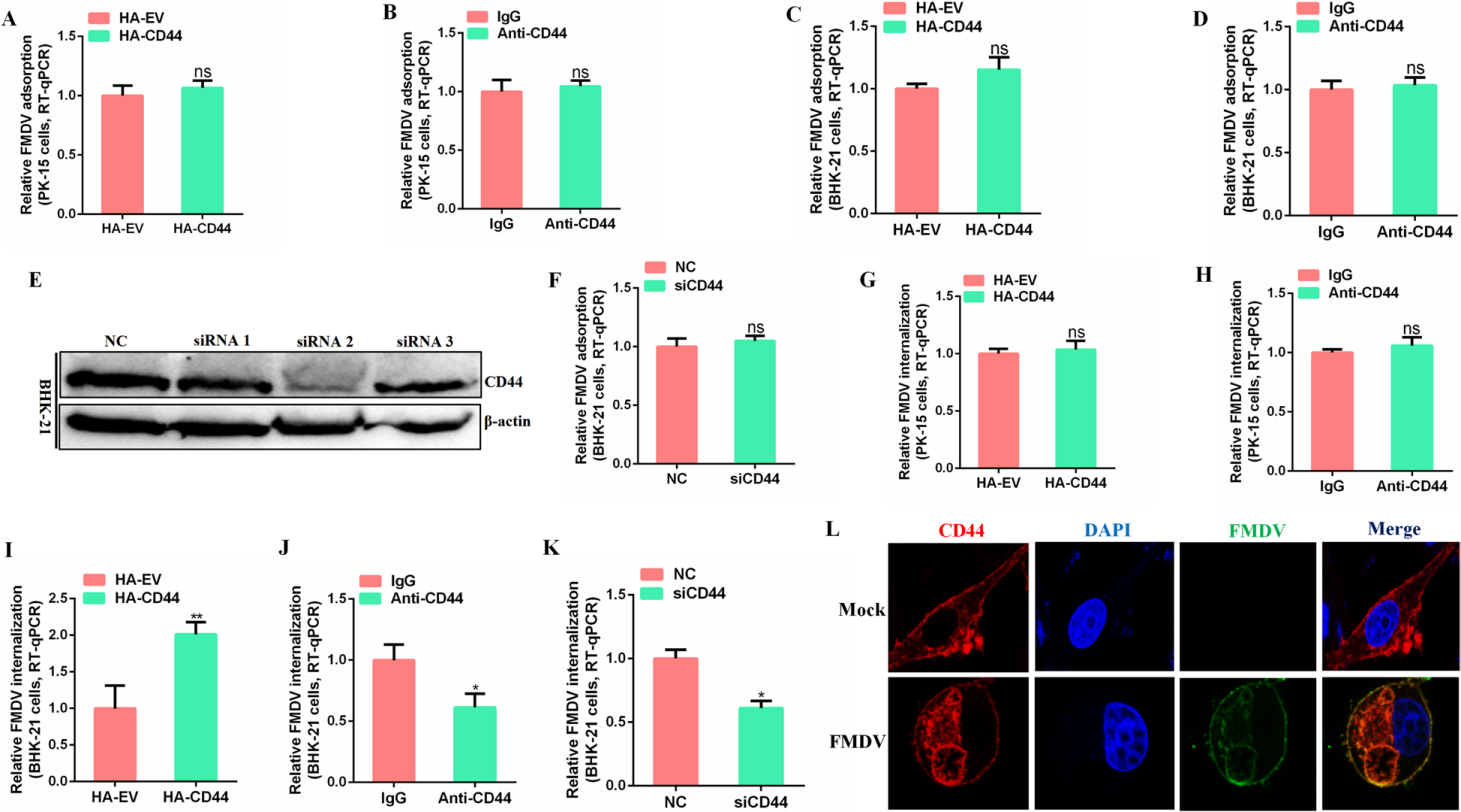
**CD44 is required for FMDV internalization in BHK-21 cells.** **A**, **C** PK-15 cells (**A**) or BHK-21 cells (**C**) transfected with plasmids encoding CD44 were infected with FMDV (10 MOI) at 4 ℃ for 1 h. After that, cells were washed and subjected to RT-qPCR. (B and D) PK-15 cells (**B**) or BHK-21 cells (**D**) pretreated with 10 μg/mL rabbit anti-CD44 antibody or rabbit IgG were infected for 1 h at 4 °C, and FMDV adsorption was determined by an RT-qPCR assay. **E** BHK-21 cells transfected with siRNA targetting CD44 were collected and assigned to western blot. **F** BHK-21 cells transfected with siRNA targeting CD44 were infected with FMDV (10 MOI) at 4 ℃ for 1 h. Cells were then washed and subjected to an RT-qPCR. (G and I) PK-15 cells **G** or BHK-21 cells (**I**) transfected with plasmids encoding CD44 were infected with FMDV (10 MOI) at 4 ℃ for 1 h. After that, cells were washed and shifted to 37 ℃ for 1 h. Cells were then treated with trypsin or proteinase K, and subjected to RT-qPCR. (**H**, **J**) PK-15 cells (**H**) or BHK-21 cells (**J**) pretreated with 10 μg/mL rabbit anti-CD44 antibody or rabbit IgG were infected with FMDV, and FMDV internalization was determined by an RT-qPCR assay. **K** BHK-21 cells transfected with siRNA targeting CD44 were infected with FMDV, and FMDV internalization was determined by an RT-qPCR assay. **L** BHK-21 cells transfected with plasmids encoding CD44 were infected with FMDV (10 MOI) for 1 h at 37 ℃, cells were then fixed, and localisation was determined using confocal microscopy. Data are means and SD of the results of three independent experiments. *P < 0.05; **P < 0.01; ns: not significant.

A previous study demonstrated that the protein level of CD44 is significantly upregulated during FMDV infection in BHK-21 cells. Given that CD44 is a membrane protein, we hypothesised that it may play a role in the attachment or internalization of the virus. The effect of CD44 on FMDV attachment was evaluated using RT-qPCR. As shown in Figures 1A, B, neither the overexpression of CD44 nor the blocking of CD44 with an anti-CD44 antibody influenced FMDV attachment in PK-15 cells. Similarly, in BHK-21 cells, overexpression of CD44 and anti-CD44 antibody blocking did not affect FMDV attachment (Figures 1C, D). Additionally, the knockdown of CD44 (Figure 1E) had no impact on FMDV attachment in BHK-21 cells either (Figure 1F).

Next, we assessed the role of CD44 in FMDV internalization using RT-qPCR. The results indicated that neither CD44 overexpression nor anti-CD44 antibody blocking influenced FMDV internalization in PK-15 cells (Figures 1G, H). Conversely, the gain of function of CD44 significantly enhanced FMDV internalization in BHK-21 cells (Figure 1I). In contrast, both anti-CD44 antibody blocking and loss of function of CD44 markedly inhibited FMDV internalization in BHK-21 cells (Figures 1J, K).

To understand the mechanism by which CD44 is involved in FMDV internalization, we investigated the kinetics of CD44 during the early phase of FMDV infection. Interestingly, we found that CD44 could internalize into cells in response to FMDV entry, whereas it predominantly remained on the cell membrane in control cells (Figure 1L).

### Inhibitor of macropinocytosis hinders CD44 internalization and affects the entry and infection of type O FMDV

**Figure 2:**
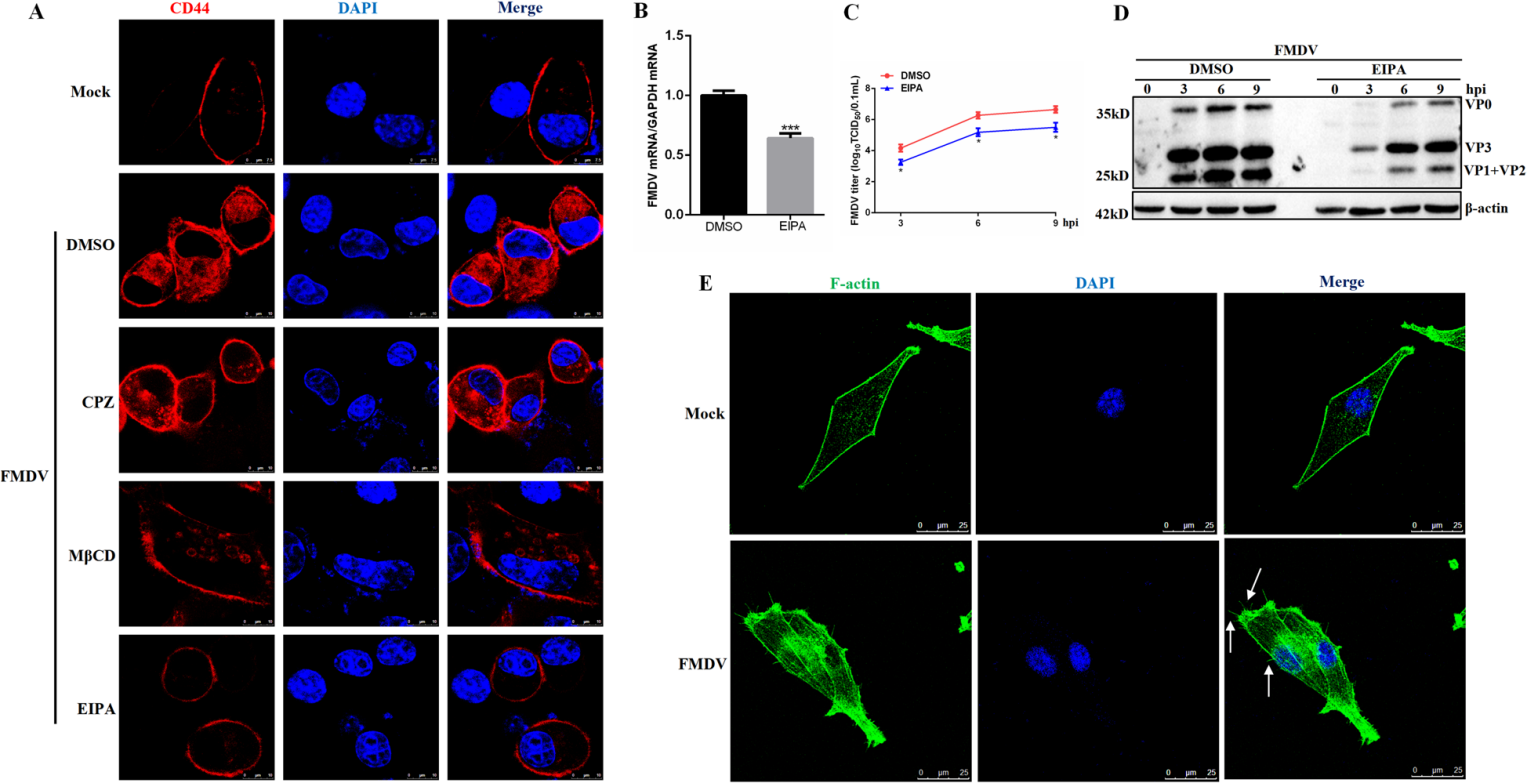
**Inhibitor of macropinocytosis impairs CD44 internalization and type O FMDV entry.** **A** BHK-21 cells transfected with plasmids encoding CD44 for 24 h were pre-treated with DMSO, EIPA (40 μM), CPZ (20 μM) or MβCD (1 mM) for 1 h, then infected with FMDV (10 MOI) at 37 ℃ in the presence of the inhibitors. At 1 h post-infection, cells were fixed and incubated with anti-HA antibodies and then with secondary antibodies conjugated with TRITC (red). Nuclei were counterstained with DAPI (blue), and localization was determined using confocal microscopy. **B** BHK-21 cells were pre-treated with DMSO or EIPA (40 μM) for 1 h, then infected with FMDV (10 MOI) at 37 ℃ in the presence of the inhibitor. At 1 h post-infection, the cell samples were collected and analysed by RT-qPCR assay to determine the viral mRNA. **C**, **D** BHK-21 cells were pre-treated with DMSO or EIPA (40 μM) for 1 h and then assigned to FMDV (1 MOI) for the indicated time points in the presence of the inhibitor. The cell lysates and supernatants were collected, and viral yields were determined by a TCID_50_ assay (**C**). The cells were collected and analysed by western blot for FMDV protein level (**D**). **E** BHK-21 cells were mock-infected or infected with FMDV (10 MOI) at 4 ℃ for 1 h. After that, cells were washed with cold PBS to remove unbound particles and the inoculum was replaced and incubated for 60 min at 37 °C. Cells were fixed and stained with 488-phalloidin, then subjected to confocal microscopy. Data are means and SD of the results of three independent experiments. *P < 0.05; ***P < 0.001.

Having observed that FMDV's entry stimulates the internalization of CD44, we next investigated whether this internalization could be blocked by inhibitors of viral entry. To our surprise, we found that 5-(N-ethyl-N-isopropyl)amiloride (EIPA), an inhibitor of macropinocytosis, significantly impaired the internalization of CD44. In contrast, chlorpromazine (which inhibits clathrin-mediated endocytosis) and methyl-β-cyclodextrin (a cholesterol-deleting compound) did not affect the internalization of CD44 (Figure 2A).

Additionally, EIPA significantly reduced the entry of type O FMDV (Figure 2B) and its replication (Figures 2C, D), indicating that type O FMDV utilises macropinocytosis as an alternative strategy to infect cells. To further investigate the role of macropinocytosis in type O FMDV entry, we examined the formation of irregular ruffles and blebs on the cell surface—features characteristic of macropinocytosis [1, 28, 29]—by staining actin filaments with 488-phalloidin. The results demonstrated that viral infection induces significant macropinocytotic features, while control cells exhibited no such phenomenon (Figure 2E). These data suggest that CD44 may play a role in the macropinocytosis-dependent entry of FMDV.

### CD44 promotes FMDV internalization via macropinocytosis

**Figure 3:**
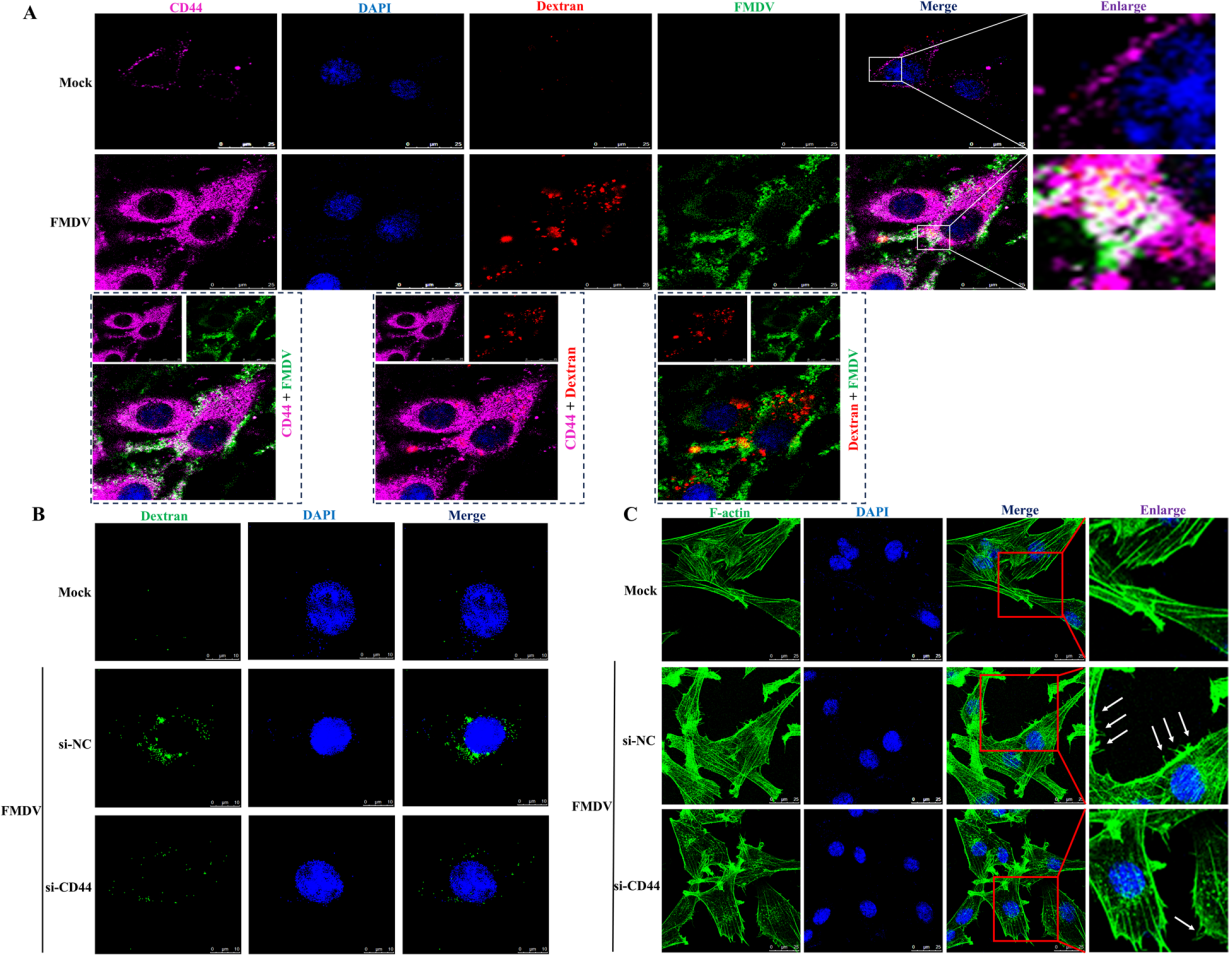
**CD44 promotes FMDV internalization via macropinocytosis**. **A** BHK-21 cells were transfected with plasmids encoding HA-CD44. At 24 h post-transfection, cells were mock-infected and infected with FMDV (10 MOI) at 4 ℃ for 1 h. After that, cells were washed with cold PBS to remove unbound particles and the inoculum was replaced with medium containing dextran 10 K-Alexa Fluor 594 and incubated for 20 min at 37 °C, cells were then fixed and incubated with anti-FMDV and anti-HA antibodies and then with secondary antibodies conjugated with FITC (green) and Alexa Fluor 647 (pink). Nuclei were counterstained with DAPI (blue), and localization was determined using confocal microscopy. **B** BHK-21 cells were transfected with CD44 siRNA. At 36 h post-transfection, cells were mock-infected or infected with FMDV (10 MOI) at 4 ℃ for 1 h. After that, cells were washed with cold PBS to remove unbound particles and the inoculum was replaced with medium containing FITC-dextran and incubated for 60 min at 37 °C. Cells were then fixed with 4% paraformaldehyde. Dextran uptake was measured by confocal microscopy. **C** BHK-21 cells were transfected with CD44 siRNA. At 36 h post-transfection, cells were mock-infected or infected with FMDV (10 MOI) at 4 ℃ for 1 h. After that, cells were washed with cold PBS to remove unbound particles and the inoculum was replaced and incubated for 60 min at 37 °C. Cells were fixed and stained with 488-phalloidin, then subjected to confocal microscopy.

To evaluate whether CD44 is involved in the macropinocytotic uptake of FMDV, we monitored the internalization of FMDV using dextran 10 K-Alexa Fluor 594, a fluorescent-labelled macropinocytosis marker [30], along with CD44. Compared to mock-infected cells, we found that CD44 and dextran colocalised with internalized FMDV (Figure 3A). These observations suggest that CD44 plays a role in the macropinocytotic entry of FMDV.

To further investigate the function of CD44 this process, we conducted an FITC-dextran uptake assay. As shown in Figure 3B, CD44 knockdown significantly reduced FMDV-induced uptake of FITC-dextran. Additionally, we examined macropinocytotic features by staining actin filaments with 488-phalloidin. The results revealed that the irregular ruffles and blebs on the cell surface were diminished in the CD44 knockdown cells compared to the control cells (Figure 3C). These findings indicate that CD44 enhances FMDV internalization by promoting macropinocytotic uptake.

### CD44 colocalises with rabankyrin-5 during FMDV internalization

**Figure 4:**
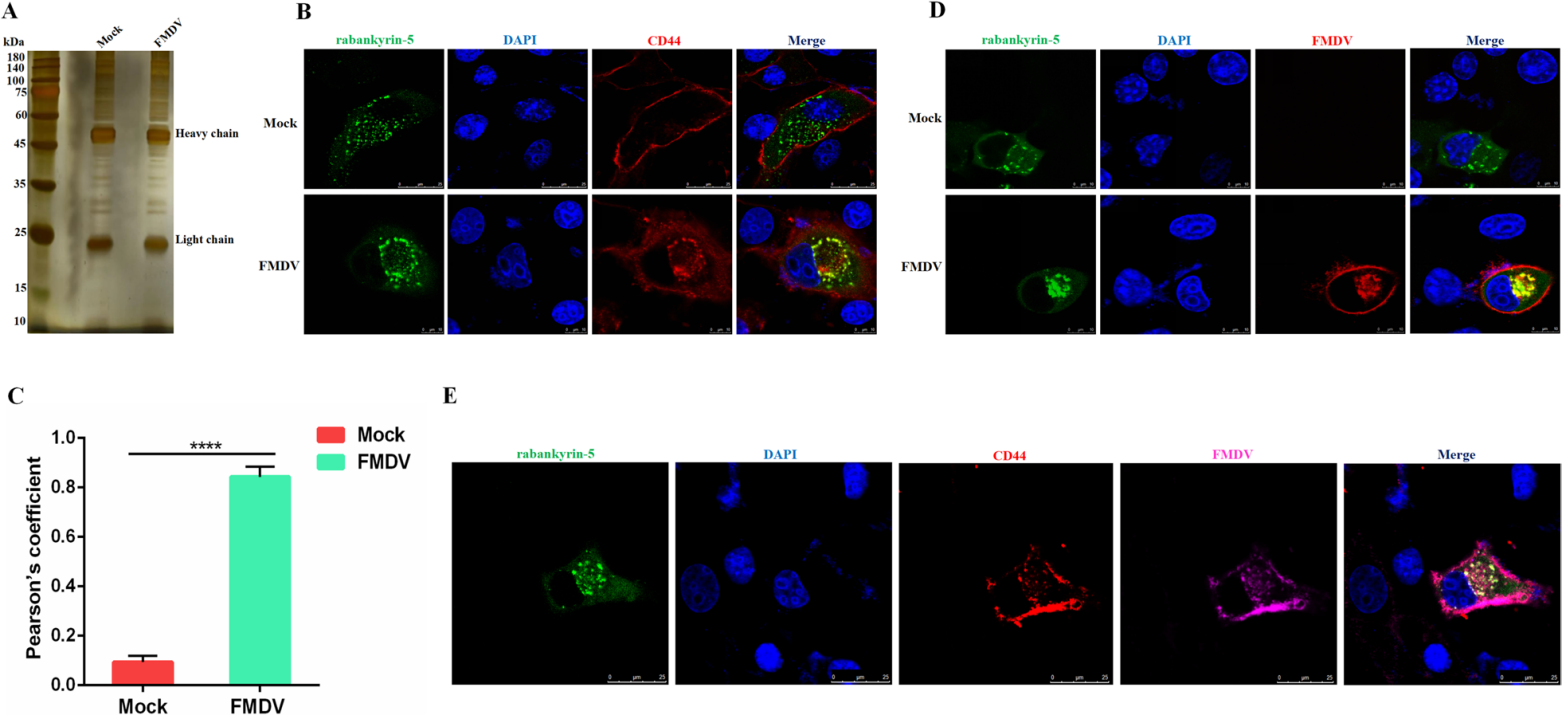
**CD44 colocalises with rabankyrin-5 during FMDV internalization.** **A** Cells mock-infected or infected with FMDV were lysed, and the cell lysates were immunoprecipitated with anti-CD44 antibody. The co-IP samples were then loaded to the SDS PAGE gel and subjected to silver staining. **B** BHK-21 cells transfected with EGFP-Rabankyrin-5 and HA-CD44 for 24 h were mock-infected or infected with FMDV (10 MOI) for 1 h at 37 °C. Cells were then fixed and incubated with anti-HA antibody and then with secondary antibodies conjugated with TRITC (red). Nuclei were counterstained with DAPI (blue), and localisation was determined using confocal microscopy. **C** The colocalisation of Rabankyrin-5 and CD44 was shown by Pearson’s coefficient. **D** BHK-21 cells transfected with EGFP-Rabankyrin-5 were mock-infected or infected with FMDV (10 MOI) for 1 h at 37 °C. The cells were then fixed and incubated with anti-FMDV serum and then with secondary antibodies conjugated with TRITC (red). Nuclei were counterstained with DAPI (blue), and localisation was determined using confocal microscopy. **E** BHK-21 cells transfected with EGFP-Rabankyrin-5 and HA-CD44 for 24 h were infected with FMDV (10 MOI) for 1 h at 37 °C. The cells were then fixed and incubated with anti-HA antibody and anti-FMDV serum, and then with secondary antibodies conjugated with TRITC (red) and Alexa Fluor 647 (pink). Nuclei were counterstained with DAPI (blue), and localisation was determined using confocal microscopy. Data are means and SD of the results of three independent experiments. ****P < 0.0001.

We conducted a co-IP and mass spectrometry assay to further validate the role of CD44 in the macropinocytotic entry induced by FMDV. Cells that were either mock-infected or infected with FMDV were lysed, and the cell lysates were immunoprecipitated using an anti-CD44 antibody. The co-IP samples were then loaded onto an SDS PAGE gel and subjected to silver staining (Figure 4A). Subsequently, the proteins separated in the gel were analysed using mass spectrometry. The Venn diagram indicated that FMDV infection significantly increased the number of proteins that interact with CD44 (Additional file 1). Among these host proteins, rabankryin-5, a known marker of macropinocytosis [31] was identified.

Next, we performed an indirect immunofluorescence assay, which confirmed that rabankyrin-5 colocalises with CD44 during FMDV internalization (Figure 4B). Furthermore, Pearson’s correlation analysis demonstrated a significant colocalisation of rabankyrin-5 with CD44, supporting our previous findings (Figure 4C). Notably, rabankyrin-5 also colocalised with FMDV (Figure 4D), and both CD44 and FMDV colocalised with rabankyrin-5 (Figure 4E). These findings provide further evidence of the involvement of CD44 in the macropinocytotic uptake of FMDV.

### The processes of CD44-mediated FMDV macropinocytotic entry

We further explored the mechanism by which CD44 initiates the macropinocytotic uptake of FMDV from two angles: (1) identifying the viral structural protein that interacts with CD44, and (2) determining whether CD44 activates PAK1, a crucial component for FMDV’s macropinocytotic entry. Given that FMDV structural proteins may engage with cellular factors during entry, and considering that CD44 plays a role in the entry of FMDV, we hypothesised that CD44 might interact with the viral structural proteins.

**Figure 5:**
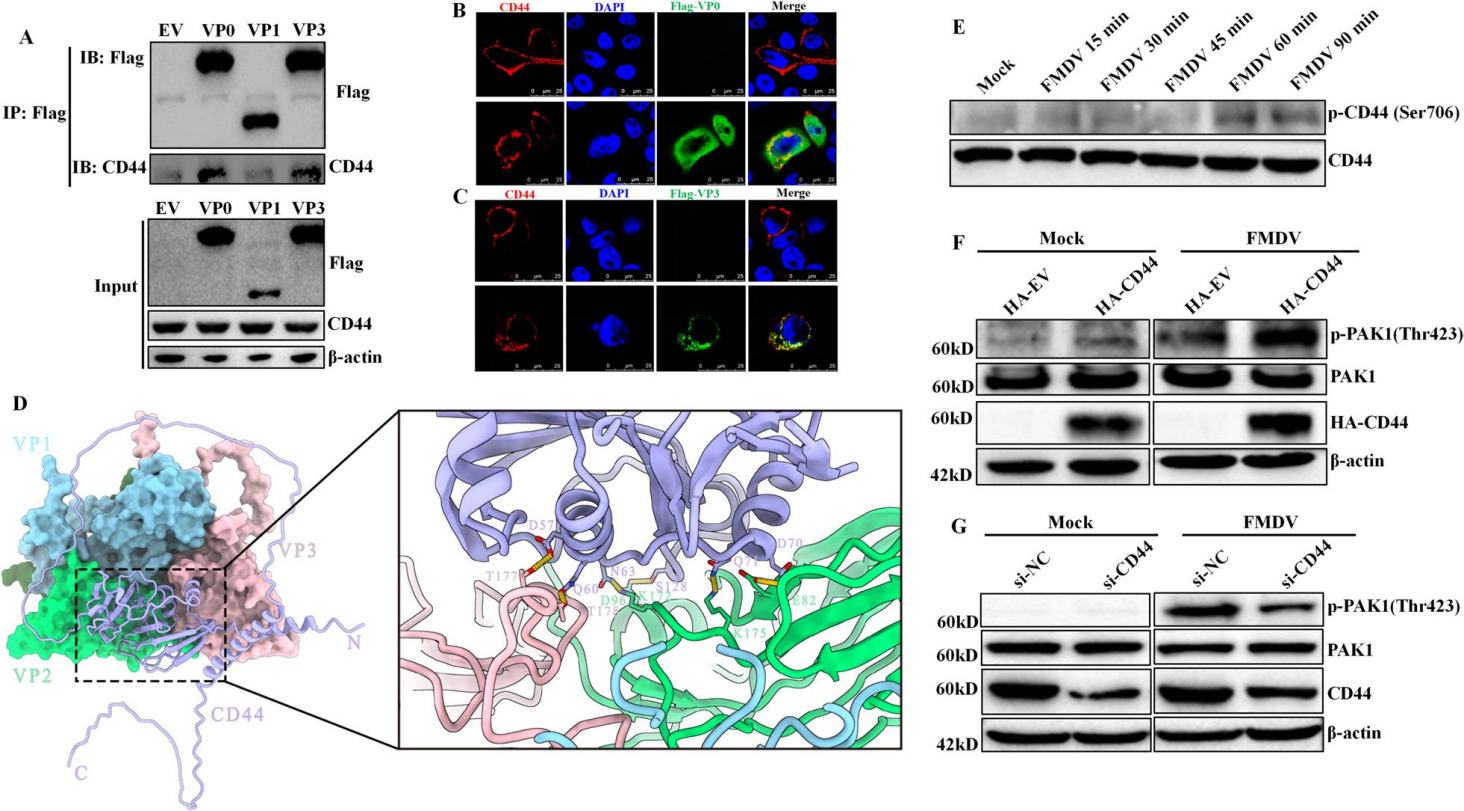
**The process of CD44-mediated FMDV macropinocytotic entry.** **A** BHK-21 cells were transfected with plasmids encoding FLAG-VP0, FLAG-VP1 and FLAG-VP3. At 24 hpt, cells were lysed on ice for 1 h and centrifuged at 15 000 × *g* for 20 min at 4 °C. Cell debris was discarded, and the supernatants were immunoprecipitated with anti-FLAG antibodies at 4 °C overnight. The immune complexes were incubated with protein G-agarose beads for 2 h, washed 5 times with lysis buffer, and eluted in SDS-PAGE buffer, then assigned to western blot analysis using anti-FLAG and anti-CD44 antibodies. **B**, **C** BHK-21 cells were transfected with plasmids encoding FLAG-VP0 (**B**) or FLAG-VP3 (**C**) and HA-CD44. At 24 hpt, cells were fixed and incubated with anti-FLAG and anti-HA antibodies and then with secondary antibodies conjugated with FITC (green) and TRITC (red), respectively. Nuclei were counterstained with DAPI (blue), and localization was determined using confocal microscopy. **D** Molecular docking model of CD44 with FMDV virion. FMDV capsid proteins are coloured with cyan (VP1), green (VP2), and pink (VP3), among which VP2 and VP3 interact with CD44, displayed with purple (left panel). The amino acid residues of the capsid protein interacting with CD44 are indicated (right panel). **E** BHK-21 cells were infected with FMDV at an MOI of 10 for the noted timepoints. The indicated protein expression levels were determined by western blot. **F** CD44 overexpressed exogenously in BHK-21 cells was mock-infected and infected with FMDV at an MOI of 10 for 60 min. The indicated protein expression levels were detected by western blot. **G** BHK-21 cells were transfected with CD44 siRNA. At 36 h post-transfection, cells were mock-infected and infected with FMDV at an MOI of 10 for 60 min. The indicated protein expression levels were detected by western blot.

To investigate this, BHK-21 cells that were transfected with plasmids encoding the proteins VP0, VP1 and VP3 were subjected to a co-IP assay to evaluate the interaction between these proteins and endogenous CD44. As shown in Figure 5A, CD44 displayed a significant interaction with the FMDV proteins VP0 and VP3. In contrast, VP1, which is recognized as the primary capsid protein of FMDV and serves as a link between FMDV and cellular integrin receptors, did not associate with CD44. This suggests that while CD44 may not be a direct receptor for FMDV, it may work in conjunction with FMDV receptors to facilitate viral entry.

We also observed that CD44 colocalises with FMDV VP0 and VP3 (Figures 5B, C). VP0 undergoes maturation cleavage, resulting in the formation of VP2 and VP4, while VP1, VP2, and VP3 are located on the exterior of the capsid, and VP4 is found on the interior. These results suggest that CD44 directly associates with the FMDV capsid proteins VP2 and VP3. To confirm this association, we conducted a molecular docking using docking simulation. Surprisingly, the docking simulation model indicated that CD44 binds to both VP2 and VP3 of FMDV (Figure 5D), thereby validating the interaction between CD44 and FMDV capsid proteins VP2 and VP3. The specific interaction residues are shown in Additional files 2 and 3.

We assessed whether CD44 is activated through its association with FMDV. Fortunately, our results indicated that CD44 undergoes phosphorylation during FMDV entry (Figure 5E). Previously, we demonstrated that FMDV entry requires the phosphorylation of PAK1, which is a critical component for macropinocytosis during FMDV infection. Consequently, we investigated whether CD44 plays a role upstream of PAK1 phosphorylation during FMDV entry.

We evaluated PAK1 phosphorylation in infected cells transfected with plasmids encoding HA-CD44 or with siRNA targeting CD44. Intriguingly, in the infected cells transfected with HA-CD44, PAK1 phosphorylation was significantly upregulated compared to cells transfected with HA-EV (Figure 5F). Conversely, the knockdown of CD44 considerably impaired the phosphorylation of PAK1 in FMDV-infected cells (Figure 5G).

Collectively, these data suggest that CD44 mediates FMDV-induced activation of PAK1 during viral entry.

## Discussion

Adsorption and internalization are critical initial steps in virus infection and propagation, heavily depending on the interaction between the virus and specific cellular functional receptors [32].

Previous studies have shown that field isolates of FMDV utilise a diversity of RGD-dependent integrin receptors, such as αvβ1, αvβ3, αvβ6, and αvβ8, to enter cells through the CME pathway [33–36]. In contrast, cell culture-adapted isolates can bind to heparan sulfate and enter cells via a caveolin-mediated endocytic pathway [37].

Additionally, macropinocytotic entry has been identified as a significant endocytosis pathway that FMDV uses to infect both PK-15 host cells and BHK-21 cells used for inactivated vaccine production. This process requires the involvement of various proteins, including Rho GTPases (specifically Rac1), Na^+^/H^+^ exchangers, several kinases (such as PAK1 and protein kinase C), myosin II, dynamin-2, and receptor tyrosine kinases [5]. However, many of the remaining signalling molecules that regulate viral macropinocytotic entry remain to be fully identified.

CD44 is a complex transmembrane glycoprotein that plays a crucial role in regulating various vital signalling pathways, thereby contributing to several biological processes. It interacts with ligands and signalling receptors, particularly receptor tyrosine kinase and non-receptor tyrosine kinases, found in the cell membrane and cell plasma. Some of these interactions are with hyaluronan [38], c-Src kinase [39], and discoidin domain receptors 1 [40], enabling CD44 to function as a signalling hub.

CD44 is involved in both physiological and pathological processes, including cell migration [9, 10], adhesion [11–13], proliferation [14], tumorigenesis, and viral infection [41]. Previous studies have shown that CD44 is essential for the life cycles of various pathogens, such as HCV, HIV-1, and HBV, facilitating their infection [42–44]. More recently, research has indicated that CD44 is also associated with FMDV infection [23, 24].

Interestingly, CD44 participates in processes that are characteristic of macropinocytosis in certain pathogen-infected cells, including cytoskeletal rearrangement and the formation of membrane ruffles [22]. However, it is still unclear whether CD44 influences FMDV’s macropinocytotic entry and what specific downstream signalling mechanisms CD44 mediates.

Here, we first performed gain-of-function and loss-of-function assays to investigate the role of CD44 in FMDV adsorption and internalization in PK-15 cells and BHK-21 cells. The results indicated that CD44 does not play a role in the adsorption process in either PK-15 cells or BHK-21 cells. However, we found that CD44 actually accelerated the internalization of FMDV specifically in BHK-21 cells.

We subsequently focused on exploring the mechanisms behind these traits. The interaction between hosts and viruses often involves mutual regulation; as viruses impose stress on hosts, hosts can evolve strategies to counteract these effects [45–47]. Additionally, hosts may develop conditions that benefit the virus [48].

In our investigation of the kinetics of CD44 in response to FMDV infection during the early phase, we observed that CD44 can internalize into cells during FMDV entry. In contrast, in control cells, CD44 is primarily localised in the cell membrane. This finding suggests that cells modify CD44 distribution to enhance FMDV internalization.

To further understand this, we assessed whether CD44 internalisation is blocked by inhibitors of macropinocytosis, clathrin-mediated endocytosis, or lipid raft-mediated endocytosis. Surprisingly, we found that EIPA, an inhibitor of macropinocytosis, impairs the internalization of CD44 induced by FMDV. Based on this, we hypothesised that CD44 may facilitate FMDV internalization through FMDV-induced macropinocytosis.

Tracking the internalization of FMDV along with dextran 10 K-Alexa Fluor 594 and CD44, we observed that CD44 and dextran colocalise with the internalized FMDV. Notably, CD44 knockdown decreased the uptake of FITC-dextran induced by FMDV, suggesting that the enhancement of FMDV internalization mediated by CD44 is linked to increased macropinocytotic uptake.

Proteomics analysis is an essential method for exploring potential interactions between pathogens and hosts [49]. To identify host factors associated with CD44 during FMDV internalization, we conducted a co-IP/mass spectrometry assay. This revealed that rabankyrin-5, a rab5 effector known to localise to and stimulate macropinocytosis, interacts with CD44. Importantly, we demonstrated that CD44 and FMDV colocalise with rabankyrin-5, confirming that CD44 acts as a macropinocytotic entry factor for FMDV.

Encouraged by these findings, we further investigated the mechanisms by which CD44 initiates FMDV macropinocytotic entry. We focused on the FMDV elements responsible for interacting with CD44 and the downstream signalling pathways activated by CD44. Fortunately, we discovered that the structural proteins VP2 and VP3 of FMDV interact with CD44. In contrast, VP1, the primary capsid protein, is recognised as the bridge between FMDV and cellular receptors [27], did not associate with CD44, suggesting that CD44 may not be a bona fide FMDV receptor.

Interestingly, a previous study showed that CD44 interacts with the structural protein VP2 of the infectious bursal disease virus (IBDV) and can serve as a cellular receptor for IBDV [8]. CD44 is relevant to the activation of some signalling molecules, including PAK1 in some cases. We also observed that CD44 undergoes phosphorylation and activates PAK1 during FMDV entry.

**Figure 6:**
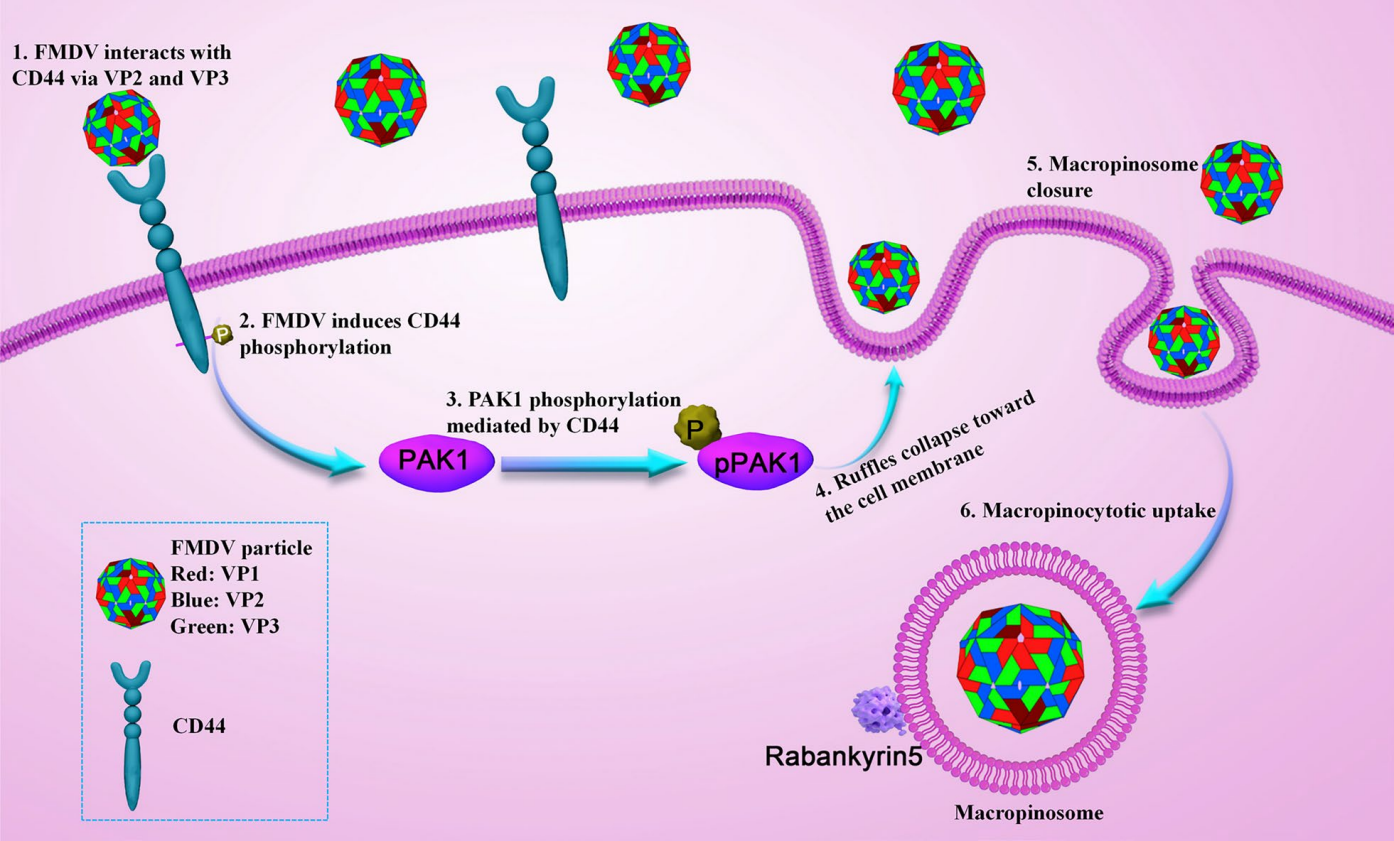
**Schematic model depicting CD44-mediated FMDV macropinocytotic entry**. CD44 interacts with FMDV VP2 and VP3 and undergoes phosphorylation during FMDV entry. Subsequently, the activated CD44 initiates the activation of PAK1, a crucial element in FMDV macropinocytotic entry, thereby promoting macropinocytosis.

In summary, our findings indicate that CD44 interacts with FMDV VP2 and VP3 and becomes phosphorylated during FMDV entry. This activation of CD44 subsequently triggers the activation of PAK1, which is a crucial component in the macropinocytotic entry of FMDV, thus facilitating macropinocytosis (Figure 6).

## Supplementary Information

**Additional file 1: Venn diagram shows the number of CD44 interaction proteins in mock control cells and FMDV-infected cells. **(A) The mass spectrometry results were analysed by Venny 2.1 online.

**Additional file 2: Prediction of interaction sites between CD44 and FMDV VP2.**

**Additional file 3: Prediction of interaction sites between CD44 and FMDV VP3.**

## Data Availability

All data generated or analysed during this study are included in this published article and its supplementary information files.
