# Supplementary figures and images for: CD44 mediates the internalization of foot-and-mouth disease virus through macropinocytosis
Source: Vet Res. 2025 Jun 21;56:123. doi: 10.1186/s13567-025-01555-3 (PMC12181885; doi:10.1186/s13567-025-01555-3)

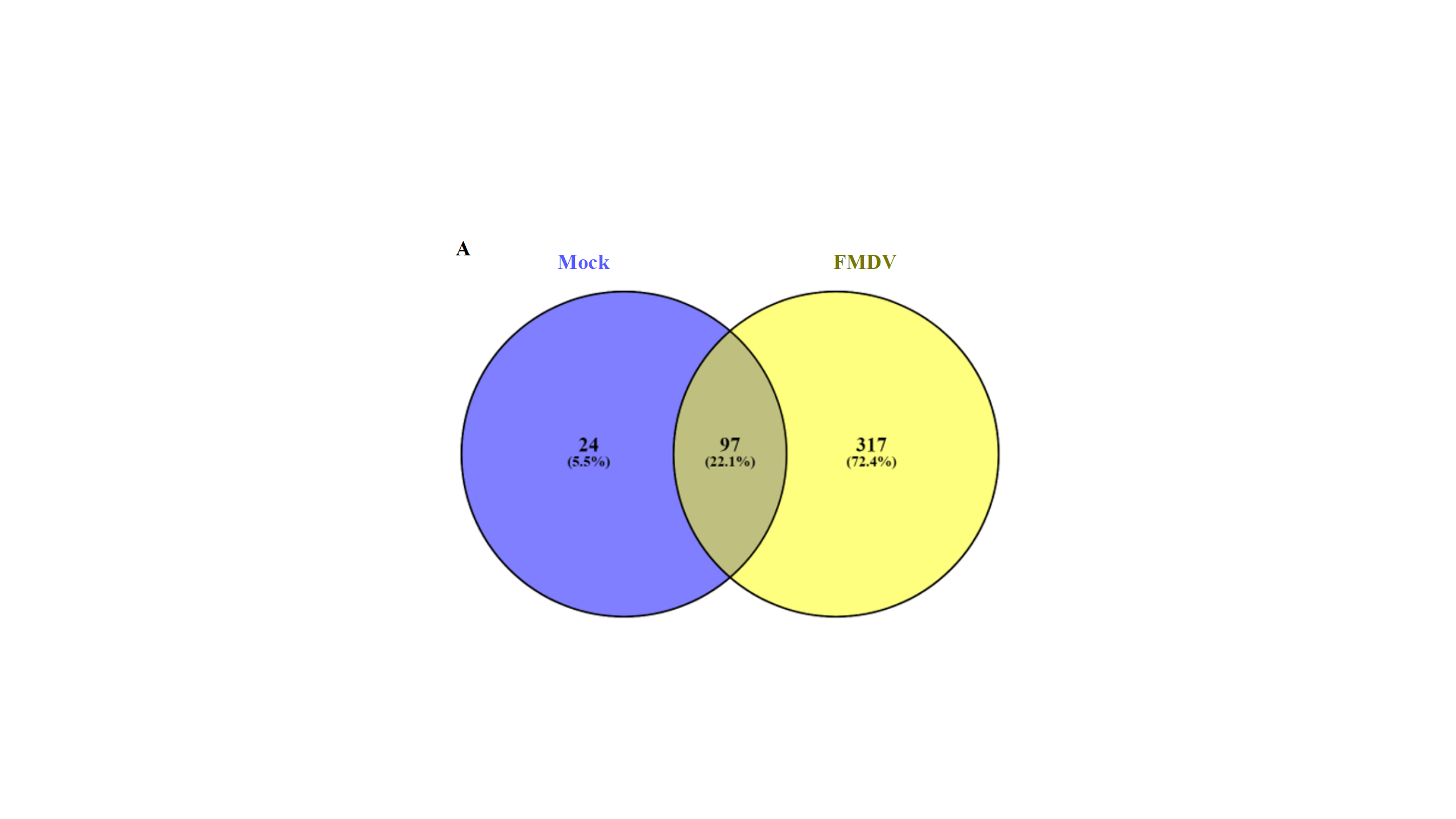

Supplement: Supplementary file 1 — Additional file 1: Venn diagram shows the number of CD44 interaction proteins in mock control cells and FMDV-infected cells. (A) The mass spectrometry results were analysed by Venny 2.1 online. [file 13567_2025_1555_MOESM1_ESM.tif]
