# Supplementary material for: CD44 mediates the internalization of foot-and-mouth disease virus through macropinocytosis
Source: Vet Res. 2025 Jun 21;56:123. doi: 10.1186/s13567-025-01555-3 (PMC12181885; doi:10.1186/s13567-025-01555-3)
Supplement: Supplementary file 2 — Additional file 2: Prediction of interaction sites between CD44 and FMDV VP2. [file 13567_2025_1555_MOESM2_ESM.docx]

**Additional file 2.** **Prediction of interaction sites between CD44 and FMDV VP2.**

| FMDV VP2 | CD44 |  |  |  |  |  |  |
| --- | --- | --- | --- | --- | --- | --- | --- |
| L80 | D70 |  |  |  |  |  |  |
| L81 | M74 |  |  |  |  |  |  |
| E82 | T68 | D70 | Q71 | M74 |  |  |  |
| T85 | L131 |  |  |  |  |  |  |
| D86 | G38 | F125 | N126 | A127 | A129 | P130 | L131 |
| T95 | S128 |  |  |  |  |  |  |
| D96 | S128 |  |  |  |  |  |  |
| L129 | D70 |  |  |  |  |  |  |
| C130 | D70 |  |  |  |  |  |  |
| S131 | F200 |  |  |  |  |  |  |
| K172 | N63 | T65 |  |  |  |  |  |
| V173 | T65 |  |  |  |  |  |  |
| K175 | Q71 | F125 |  |  |  |  |  |
