# Supplementary material for: CD44 mediates the internalization of foot-and-mouth disease virus through macropinocytosis
Source: Vet Res. 2025 Jun 21;56:123. doi: 10.1186/s13567-025-01555-3 (PMC12181885; doi:10.1186/s13567-025-01555-3)
Supplement: Supplementary file 3 — Additional file 3: Prediction of interaction sites between CD44 and FMDV VP3. [file 13567_2025_1555_MOESM3_ESM.docx]

**Additional file 3.** **Prediction of interaction sites between CD44 and FMDV VP3.**

| FMDV VP3 | CD44 |  |  |  |
| --- | --- | --- | --- | --- |
| G129 | N63 | N143 |  |  |
| M130 | N63 | N143 |  |  |
| E131 | P142 | N143 | S144 | F145 |
| A174 | H170 |  |  |  |
| A175 | H170 |  |  |  |
| T177 | D57 | T169 | H170 |  |
| T178 | Q60 |  |  |  |
